# Age-related gray matter volume changes in the brain during non-elderly adulthood

**DOI:** 10.1016/j.neurobiolaging.2009.02.008

**Published:** 2011-02

**Authors:** Débora Terribilli, Maristela S. Schaufelberger, Fábio L.S. Duran, Marcus V. Zanetti, Pedro K. Curiati, Paulo R. Menezes, Márcia Scazufca, Edson Amaro, Cláudia C. Leite, Geraldo F. Busatto

**Affiliations:** a Laboratory of Psychiatric Neuroimaging (LIM-21), Department of Psychiatry, University of São Paulo Medical School, São Paulo, Brazil; b Department of Preventive Medicine, University of São Paulo Medical School, São Paulo, Brazil; c Department of Radiology, University of São Paulo Medical School, São Paulo, Brazil

**Keywords:** Prefrontal cortex, Insula, Limbic system, Gray matter, Aging, Gender, Voxel-based morphometry, Magnetic resonance imaging

## Abstract

Previous magnetic resonance imaging (MRI) studies described consistent age-related gray matter (GM) reductions in the fronto-parietal neocortex, insula and cerebellum in elderly subjects, but not as frequently in limbic/paralimbic structures. However, it is unclear whether such features are already present during earlier stages of adulthood, and if age-related GM changes may follow non-linear patterns at such age range. This voxel-based morphometry study investigated the relationship between GM volumes and age specifically during non-elderly life (18–50 years) in 89 healthy individuals (48 males and 41 females). Voxelwise analyses showed significant (*p* < 0.05, corrected) negative correlations in the right prefrontal cortex and left cerebellum, and positive correlations (indicating lack of GM loss) in the medial temporal region, cingulate gyrus, insula and temporal neocortex. Analyses using ROI masks showed that age-related dorsolateral prefrontal volume decrements followed non-linear patterns, and were less prominent in females compared to males at this age range. These findings further support for the notion of a heterogeneous and asynchronous pattern of age-related brain morphometric changes, with region-specific non-linear features.

## 1 Introduction

In recent decades, the use of high-resolution magnetic resonance imaging (MRI) has allowed detailed assessments of the morphometric brain changes associated with aging in the living human brain. These MRI studies have either quantified regional brain volumes using manually defined regions-of-interest (ROI) placed on selected brain structures (Mu et al., 1999; Jernigan et al., 2001; Raz et al., 2003), or used automatized, voxel-based methods for the testing of age-related anatomical differences across the entire cerebral volume (Good et al., 2001; Grieve et al., 2005; Kalpouzos et al., 2009; Lemaitre et al., 2005). The latter techniques include the voxel-based morphometry (VBM) approach (which allows voxelwise gray and white matter volume analyses) (Good et al., 2001), and the deformation-field-morphometry (DFM) methodology (which enables automated quantifications of differences in brain shape) (Pieperhoff et al., 2008).

Using the above methods, several MRI studies have reported a consistent pattern of age-related gray matter (GM) volumetric reductions in the human neocortex, involving mainly prefrontal regions as well as the parietal and temporal association cortices (Jernigan et al., 2001; Sowell et al., 2001, 2003; Resnick et al., 2003; Tisserand et al., 2000, 2002). In addition, there have been relatively consistent reports of age-related GM deficits in the insula, cerebellum, basal ganglia and thalamus (Good et al., 2001; Taki et al., 2004; Grieve et al., 2005; Alexander et al., 2006; Kalpouzos et al., 2009; Abe et al., 2008). These studies have used either cross-sectional designs investigating correlations between brain volumes and age (Jernigan et al., 2001), or longitudinal approaches with repeated measurements in the same subjects over time (Resnick et al., 2003).

Conversely, findings regarding age-related volumetric changes in limbic and paralimbic regions have been conflicting. A few morphometric MRI studies have reported trends towards age-related atrophy in the amygdala, hippocampus and cingulate gyrus of elderly individuals. However, other MRI investigations have suggested that the volumes of temporolimbic and cingulate brain regions do not show volumetric changes during the aging process (Sullivan et al., 1995; Good et al., 2001; Taki et al., 2004; Grieve et al., 2005; Alexander et al., 2006; Kalpouzos et al., 2009; Abe et al., 2008). The latter findings have provided support for the notion that neocortical regions that mature later in the human brain are more susceptible to age-related morphologic changes.

A summary of all VBM studies of age-related GM changes in healthy subjects to date is provided in Table 1. Given the relatively contradictory findings of those studies, further investigations are needed to confirm the hypothesis that early-maturing, subcortical regions are less vulnerable to gray matter reductions during the aging process of the human brain. The understanding of this process is a relevant issue, given the prominent role of limbic and paralimbic regions to a wide range of emotional and cognitive functions (Phillips et al., 2003a; Gallagher and Schoenbaum, 1999; Kumaran and Maguire, 2005; Pribram, 2006).

Most cross-sectional morphometric MRI studies of healthy aging have used linear methods to investigate relationships between age and regional GM indices. However, it is possible that such relationships follow variable, non-linear patterns across separate brain portions (Allen et al., 2005; De Carli et al., 2007; Grieve et al., 2005), and this could partially explain discrepancies in the results of previous MRI studies of healthy aging. In the first morphometric MRI study that addressed such issue, involving a sample of 87 healthy individuals aged between 22 and 88 years, Allen et al. (2005) used a ROI-based approach to investigate age-related gray and white matter volume changes across the cerebral cortex and hippocampal region. They found that the brain region where volume reductions were most strongly associated with aging was the frontal GM; also, they reported that age-related volumetric changes in other cerebral regions, such as the hippocampus and lateral temporal cortex, were best modeled by non-linear regression models. More recently, Kennedy et al. (in press) investigated age-related brain volume changes in a large sample (*n* = 200) across a wide age span (18–81 years) using both VBM and manual, ROI-based volumetry. They reported significant inverse relationships between age and brain volumes by both methods; moreover, the ROI-based measurements of some brain regions, such as the hippocampus and prefrontal white matter, uncovered accelerated patterns of age-related volume decline that were best explained by non-linear models. Finally, these authors found that the use of VBM-derived information across voxels contained in specific, anatomically defined ROI masks, confirmed the non-linearity of patterns of age-related volume changes detected by manual volumetry (Kennedy et al., in press).

Most of the large morphometric MRI investigations reviewed above have evaluated aging effects on regional brain volumes across broad age ranges, usually from the second to the eightieth–ninetieth decades of life (Good et al., 2001; Sowell et al., 2003; Grieve et al., 2005; Allen et al., 2005; Kennedy et al., in press). Due to that, there are limited data regarding to specific differences in the rates of age-related volumetric reductions in neocortical versus limbic–paralimbic regions during adult, non-elderly life. This is a relevant issue, as several neuropsychiatric disorders that typically have their onset during adulthood are thought to involve structural abnormalities in those brain regions (Mayberg, 2003; Honea et al., 2005; Adler et al., 2007). In one recent DFM study that did recruit a sample of adults up to 51 years (*n* = 51), Pieperhoff et al. (2008) described increased vulnerability to aging in subcortical nuclei and cortical areas of the sensorimotor system, as well as a pronounced age-related decline in the anterior cingulate, orbitofrontal and lateral prefrontal cortices.

The present MRI study attempted to address the two above issues in combination. Firstly, in order to specifically investigate age effects during adult, non-elderly life, we chose to assess the presence of significant correlations between age and GM volumes in neocortical, limbic–paralimbic, insular and cerebellar regions in a sample of healthy individuals (*n* = 89) with an age range restricted to approximately three decades of elderly adult life (from 18 to 50 years of age). In contrast with previous cross-sectional MRI studies assessing GM changes during normal aging, we employed an epidemiological design to recruit our sample, with all subjects randomly selected from the same circumscribed geographical region. Secondly, in order to provide detailed information about the nature of the relationship between GM volumes and aging at this age range, we performed two types of analyses: initially, we conducted a voxelwise search for significant linear correlations and age, using the conventional VBM approach; subsequently, we conducted a VBM-derived analysis using anatomically defined ROI masks to estimate the volumes of specific brain structures across the sample, with the purpose of testing whether the relationship between GM volumes and age in each region would be best predicted by non-linear models.

With such two-step analyses, we aimed to ascertain whether it is possible to differentiate, during non-elderly life, patterns of age-related GM reductions in neocortical areas, insula and cerebellum versus lack of volumetric changes of limbic/paralimbic structures. In addition, we wished to verify whether age-related volumetric reductions in separate brain structures occur in different degrees of severity and following different linear or non-linear models.

## 2 Methods

### 2.1 Subjects

The study sample was drawn from a database of healthy subjects selected as the control group for an epidemiologically based neuroimaging study that investigated the presence of brain volumetric changes in patients experiencing their first episode of psychosis in the city of São Paulo, Brazil (Schaufelberger et al., 2007). Such healthy individuals were next-door neighbours to the psychosis subjects, and they had been living, for at least 6 months, in the same circumscribed area of São Paulo city, formed by 21 districts under the same public health administration and comprising approximately 900,000 inhabitants. They were screened according to the following exclusion criteria: (a) history of head injury with loss of consciousness; (b) presence of neurological disorders or any organic disorders that could affect the central nervous system; (c) contraindications for MRI scanning; (d) personal history of axis I psychiatric disorders, except mild anxiety disorders, as assessed by the Structured Clinical Interview for DSM-IV (SCID) (First et al., 1995); (e) substance abuse or dependence diagnosis as investigated with the SCID, the Alcohol Use Disorders Identification Test (AUDIT) (Saunders et al., 1993) and the South Westminster Questionnaire (Menezes et al., 1996).

Over the duration of the above epidemiological investigation (between 2002 and 2005) (Schaufelberger et al., 2007), a total of 114 healthy individuals from the catchment area accepted to take part in the study and underwent MRI scanning. However, 9 subjects had to be excluded from the present investigation due to the presence of silent gross brain lesions, 11 due to artifacts during image acquisition, and 5 due to substance abuse or dependence, resulting in a final sample of 89 subjects (mean age = 30.17 years, SD = 8.35). There were 41 females and 48 males. A summary of their demographic and clinical characteristics is given in Table 2.

Males and females did not differ in demographic and clinical variables, such as years of education (*t*-test = 1.30, df = 87, *p* = 0.196), familial monthly income (*t*-test = 1.79, df = 85, *p* = 0.076), socioeconomic class (*χ*^2^ = 5.09, df = 4, *p* = 0.278) and handedness (*χ*^2^ = 3.63, df = 2, *p* = 0.162). However, females were significantly older (*t*-test = 2.98, df = 87, *p* = 0.004) and more frequently married than males (*χ*^2^ = 7.11, df = 1, *p* = 0.008).

Local ethics committees approved the project, and all participants signed a consent form after being fully informed about the procedures involved in the experiment.

### 2.2 Image acquisition and processing

Imaging data were acquired using two identical scanners (1.5 T GE Signa scanner, General Electric, Milwaukee WI, USA). Exactly the same acquisition protocols were used (a T1-SPGR sequence providing 124 contiguous slices, voxel size = 0.86 mm × 0.86 mm × 1.5 mm, TE = 5.2 ms, TR = 21.7 ms, flip angle = 20, FOV = 22, matrix = 256 × 192). The subsamples investigated in scanner 1 (*n* = 53) and scanner 2 (*n* = 36) were not significantly different in regard to their mean ages (scanner 1 = 29.08 ± 7.37 years, range 18–44; scanner 2 = 31.78 ± 9.50 years, range 18–50; *t* = −1.44, *p* = 0.155) and gender distribution (scanner 1 = 29 males/24 females, scanner 2 = 19 males/17 females; (*χ*^2^ = 0.032, df = 1, *p* = 0.857). All images were visually inspected by an experienced radiologist with the purpose of identifying artifacts during image acquisition and the presence of silent gross brain lesions.

Imaging data were processed using the statistical parametric mapping (SPM2) package (Wellcome Department of Cognitive Neurology, Institute of Neurology, London, UK), executed in Matlab 5.3 version (Mathworks, Sherborn, MA), according to the SPM2 optimized protocol (Good et al., 2001). This involved, initially, the creation of a standard template set specifically for the study, consisting on an average T1-weighted image and *a priori* GM, white matter and cerebrospinal fluid templates, based on the images of all subjects (Good et al., 2001; Schaufelberger et al., 2007). Subsequently, the processing of the original images from all subjects was carried out, beginning by image segmentation with the study specific, *a priori* GM, white matter and cerebrospinal fluid templates (Good et al., 2001). Extracted GM images were then spatially normalized to the customized GM templates with 12-parameter linear and non-linear transformations (7_9_7 basis functions). The parameters resulting from this spatial normalization step were then reapplied to the original structural images. These fully normalized images were re-sliced using tri-linear interpolation to a voxel size of 2 mm × 2 mm × 2 mm and segmented into GM, white matter and cerebrospinal fluid partitions. Voxel values were modulated by the Jacobian determinants derived from the spatial normalization, thus allowing brain structures that had their volumes reduced after spatial normalization to have their total counts decreased by an amount proportional to the degree of volume shrinkage (Good et al., 2001). Finally, images were smoothed using a 12 mm Gaussian kernel, with the purpose of reducing variations caused by inter-individual differences in the anatomy of the gray and white matter brain compartments.

The reliability of the MRI data obtained with the two scanners was assessed by intra-class correlation coefficients (ICC) calculated based on estimates of regional GM, obtained using the spatially normalized volumes of interest within the Automatic Anatomical Labeling (AAL) SPM2 toolbox. Six healthy volunteers were (re)examined on the same day using both scanners. We obtained ICC values higher than 0.90 for all cortical regions and medial temporal structures, and values between 0.83 (thalamus) and 0.23 (putamen) in the subcortical nuclei, as previously reported (Schaufelberger et al., 2007).

### 2.3 Statistical analyses

Initially, we investigated the presence of significant linear correlations between age and regional GM volumes with VBM using the general linear model, based on random Gaussian field theory (Friston et al., 1994). Such voxelwise investigation was performed for the overall sample (with covariance for gender), and subsequently for the male and female subgroups separately. Only voxels with values above an absolute GM threshold of 0.05 entered the analyses. Resulting statistics at each voxel were transformed to *Z*-scores and displayed as SPMs into standardized space, at an initial threshold of *Z* > 3.09.

The above correlation analyses were conducted both with and without covariance for the global amount of GM in the brain. This confounding covariate was given by the total number of voxels within the GM compartment of each subject. The strategy of including this covariate led, on the analyses for negative correlations, to the identification of localized brain areas where age-related GM losses were greater than the overall degree of GM decrement in the brain. Conversely, the statistical maps for positive correlations, covaried for total GM, indicated the localized brain regions where GM volumes did not decrease in the same proportion as the overall degree of GM loss in the brain. Each SPM was inspected on a hypothesis-driven fashion, searching for voxel clusters in specific brain regions where significant findings were predicted *a priori*. This hypothesis-driven analysis was conducted using the small volume correction (SVC) method, with the purpose of constraining the total number of voxels included in the analyses. Each region was circumscribed by merging the spatially normalized ROI masks that are available within the AAL SPM toolbox. Seven ROI masks were used for each hemisphere, involving respectively the: prefrontal cortex (dorsomedial, dorsolateral and orbitofrontal portions); lateral parietal neocortex (supramarginal, angular and superior parietal gyri); lateral temporal neocortex (superior, middle and inferior temporal gyri); insula; temporolimbic region (amygdala, hippocampus and parahippocampal gyrus); anterior cingulate gyrus; and cerebellum. Findings of these hypothesis-driven, SVC-based analyses were reported as significant if surviving family-wise error (FWE) correction for multiple comparisons (*p* < 0.05) over the respective volume of interest, with voxel clusters comprising at least 20 voxels. The anatomic location of each resulting cluster was determined using the Talairach and Tournoux Atlas coordinates (Talairach and Tournoux, 1988), converted from the MNI system (Brett et al., 2002).

Subsequently, in order to investigate patterns of non-linearity in the relationship between GM volumes and age, regression analyses were performed with SPSS 10.0 software. Age was the fixed factor and the dependent variables were the estimates of regional GM volumes extracted from the spatially normalized images of each subject (corrected for the global amount of GM in the brain), using each of the fourteen ROI masks described above. The goodness of fit of first, second and third order polynomial expansions was assessed and results were reported only if at least one of the regression models achieved the significance level set at *p* < 0.05. All analyses were firstly performed for the overall sample of healthy individuals, and subsequently for the male and female subgroups separately.

## 3 Results

### 3.1 Voxel-based morphometry findings

**Fig. 1:**
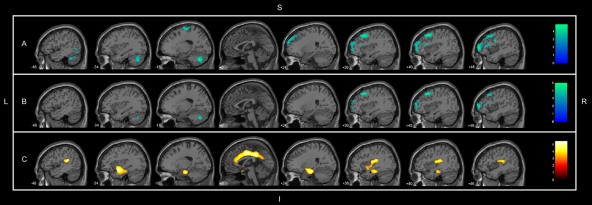
Results of the whole-brain search of significant correlations between gray matter (GM) volumes and age in the overall sample of healthy non-elderly individuals (*n* = 89) (at the *Z* > 3.09 threshold, corresponding to *p* < 0.001 and an extent threshold of 300 voxels). Foci of significance were overlaid on sagittal brain slices spatially normalized into an approximation to the Talairach and Tournoux stereotactic atlas (1988). The numbers associated with each frame represent standard coordinates in the *x* axis. (A) Foci of negative correlation without covariance for total GM volume (highlighted in blue), representing the atrophic changes that occur with the aging process. The right prefrontal cortex and left cerebellum are the most prominent areas of global brain volume reduction. (B) Areas of negative correlation with covariance for total GM volume (highlighted in blue) showed restricted areas of atrophy in the right prefrontal cortex and left cerebellum. (C) Foci of significant positive correlation with covariance for total GM volume (highlighted in yellow) indicating the brain regions where GM decrements did not occur in the same proportion as the overall degree of GM loss with aging, including: the entire extension of the cingulate gyrus, the amygdala-hippocampal complex, the parahippocampal gyrus and insula bilaterally, as well as the posterior temporal cortex and precuneus. Abbreviations*:* S, superior; I, inferior; R, right; L, left. (For interpretation of the references to color in this figure legend, the reader is referred to the web version of the article.)

In the whole sample of healthy adults, large clusters of significant (peak voxel *p*-FWE ≤ 0.05) negative correlations between age and GM volume were observed in neocortical areas of the frontal lobe, as well as in extensive portions of the anterior and posterior lobes of the left cerebellar hemisphere, mainly involving the cerebellar pyramid (Fig. 1A). There were no regions of significant positive correlations between gray matter volumes and age.

When the above voxelwise calculations were repeated with covariance for total GM volume, the SVC-based analyses demonstrated the presence of significant linear correlations (*p*-FWE ≤ 0.05) between GM volume and age in several of the brain structures where significant findings had been hypothesized *a priori* (Table 3). Foci of significant negative correlations were found in the right middle frontal gyrus and the posterior lobe of the left cerebellum (Fig. 1B). Conversely, foci of significant positive correlation between GM volume and age were detected bilaterally in the: cingulate gyrus, parahippocampal gyrus, amygdala, hippocampus, and superior temporal gyrus (Fig. 1C). Contrary to the initial prediction of the study, we also detected foci of significant positive correlation in the insula bilaterally. No significant volume changes were observed in lateral parietal and occipital regions (Fig. 1C).

The same overall pattern of results was obtained when the VBM analyses were repeated including scanner (#1 or #2) as a nuisance covariate. We also conducted VBM-based linear correlation analyses separately for each scanner (i.e. *n* = 53 and 36) with the specific aim of determining whether any significant relationship emerged in the subcortical regions where low inter-scanner ICC values had been found. In either scanner subgroup, no significant clusters of negative correlations indicative of excessive GM loss emerged in the basal ganglia or thalamus at the uncorrected *p* < 0.001 threshold. In the subsample examined with scanner 1, there were actually clusters of positive correlation at this threshold involving respectively the left caudate nucleus (38 voxels, peak coordinates = −4, 6, −8, *Z* = 4.22) and the right putamen (23 voxels, 01, peak coordinates = 32, 6, −8, *Z* = 3.37).

In the male subgroup, the voxelwise search for significant linear correlations between regional GM volumes and age, with covariance for total GM volumes, showed findings of negative and positive correlations that were broadly similar to those reported for the overall sample (Table 4). In the female subgroup, conversely, there were no regions showing significant negative or positive linear correlations between GM volumes and age.

### 3.2 Regression analyses using ROI masks

In the overall sample (*n* = 89), the rate of global GM decline was best fit by a linear model (*R*^2^ = 0.11, *p* = 0.002).

**Fig. 2:**
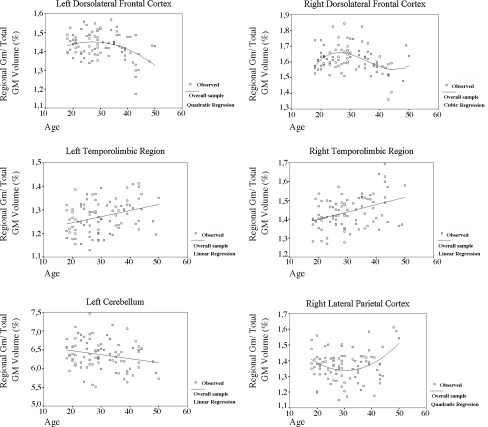
Plots of gray matter versus age (including best fit regression lines) in the overall sample healthy individuals (*n* = 89). Mean gray matter volumes for each brain region were extracted from the spatially normalized images of each subject using standardized ROI masks, and corrected for the total amount of gray matter in the brain. Only regions in which at least one regression model was significant at the *p* < 0.05 threshold are represented (see Table 5 for details).

**Fig. 3:**
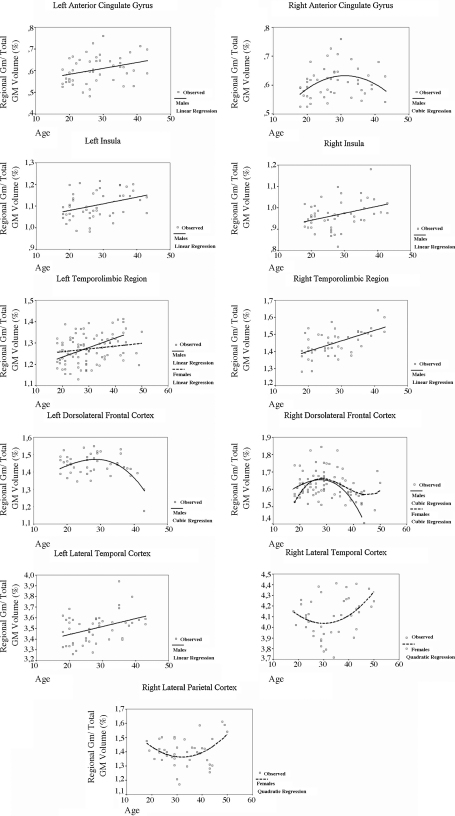
Plots of gray matter versus age (including best fit regression lines) in male (*n* = 48) and female (*n* = 41) subgroups. Mean gray matter volumes for each brain region were extracted from the spatially normalized images of each subject using standardized ROI masks, and corrected for the total amount of gray matter in the brain. Only regions in which at least one regression model was significant for male or female subgroups at the *p* < 0.05 threshold are represented (see Table 5 for details).

Results for the regression analyses of regional GM volumes (as estimated using ROI masks) versus age are presented in Table 5 and Figs. 2 and 3. There was a bilateral pattern of non-linear GM loss in the dorsolateral prefrontal cortex, with an ascending curve – representing a smaller rate of volume decrement in this region relative to the overall GM loss in the brain – until the fourth decade of life and accelerated volumetric loss from then onwards (Table 5 and Fig. 2). In the parietal cortex, however, an opposite profile was found, with volumetric decline up until the end of the third decade, followed by an ascending pattern between 30 and 50 years of age (Table 5 and Fig. 2). There were also regions in which age-related neuroanatomical changes were best represented by linear regression models: a constant rate of relative GM increase (i.e. less GM loss than the global GM decline) was found bilaterally in the temporolimbic region, while a steady GM decline was detected in the left cerebellum (Table 5 and Fig. 2). Finally, there were trends for linear correlations (*p* < 0.10) in the left lateral temporal cortex and right cerebellum that were due to a positive correlation in the former region and a steady pattern of GM loss in the latter region (Table 5).

The best fitting regression models for the separate male and female subgroups are also provided in Table 5. In males, linear positive correlations were found in the left lateral temporal cortex and anterior cingulate gyrus, and bilaterally in the insula and temporolimbic region, whereas volumetric decline, best represented by non-linear regression models, was seen in the bilateral dorsolateral prefrontal cortex and right anterior cingulate gyrus (Table 5 and Fig. 3). In females, there was a linear positive correlation in the left temporolimbic region, similarly to the pattern identified in the male subgroup (Fig. 3). However, there was no GM decline in the left dorsolateral prefrontal cortex as seen in males (Table 5), whereas the right dorsolateral prefrontal cortex displayed a pattern of GM decline explained by a cubic fit, in lesser degree than in males. Also, a quadratic regression model indicative of a decrease in regional GM vulnerability to aging between 30 and 50 years of age, emerged as the best fit in the right lateral temporal and parietal cortices only in the female subgroup (Fig. 3). Finally, trends towards non-linear regional GM decline with aging (*p* < 0.10) were found in the right orbital frontal and dorsomedial prefrontal cortices in males and in the left cerebellum in females (Table 5).

## 4 Discussion

This cross-sectional morphometric MRI study investigated the relationship between aging and GM volumes in a relatively large sample of non-elderly adult healthy subjects (from 18 to 50 years of age). To the best of our knowledge, this study is the first of its kind that employed a population-based design, recruiting individuals who were living for a stable period of time in the same circumscribed geographical area.

Our VBM-based pattern of inverse correlations between prefrontal GM volumes and age replicated the results of the three largest VBM studies carried out to date investigating the same issue (Good et al., 2001; Grieve et al., 2005; Kennedy et al., in press); these studies have used similar cross-sectional designs as described herein, but spanned a greater age range than ours. In the only VBM study that investigated the presence of age-related neocortical changes specifically in non-elderly adult subjects, Sowell et al. (1999) compared a small sample of adolescent (*n* = 10, age range 12–16) with a group of young adults (*n* = 10, age range 23–30 years), and found significant GM reductions in the latter group in dorsal, medial and lateral portions of the frontal lobes. More recently, a larger DFM study of healthy, non-elderly male subjects (*n* = 51, age range 18–51 years) also reported cortical volume decrements in association with aging (Pieperhoff et al., 2008). Taken together with these results, our findings indicate that age-related neocortical volume reductions are detectable with VBM methods during non-elderly, adult life.

In contrast with the inverse relationship between age and prefrontal cortical volumes, the whole-brain VBM analyses uncovered significant positive rather than negative correlations between the GM volumes of lateral temporal areas and age. Also, our VBM analysis showed no evidence of significant neocortical parietal volume reductions in association with aging. In the above-cited VBM investigation of age-related volume changes in non-elderly adults carried out by Sowell et al. (1999), the neocortical volumetric shrinkage seen in young adults relative to adolescents in frontal regions did not extend to the parietal and temporal lobes. Also, in the recent DFM study of non-elderly adults by Pieperhoff et al. (2008), age-related cortical volumetric reductions were restricted to frontal regions and the sensory-motor system, almost completely sparing the temporal and occipital cortices (Pieperhoff et al., 2008). Together with the latter findings, our results concur with the notion that, during non-elderly adult life, there are distinctions in the pattern of age-related GM losses in the prefrontal cortex relative to other neocortical regions (Sowell et al., 2004).

The estimation of regional brain volumes from the VBM data using ROI masks allowed us to investigate possible non-linear features of the relationship between the volumes of neocortical regions and age which would not be otherwise detectable (Kennedy et al., in press). This analysis uncovered patterns of age-related volume decline in the dorsolateral prefrontal cortex that were best fit by a quadratic model in the left hemisphere and a cubic model for the right side. These findings reflected a pattern of no GM decline until the third decade of life, with an increase in GM vulnerability to aging from then onwards. To our knowledge, such specific profile of age-related GM changes in the prefrontal cortex has not been previously reported. The selectivity of such profile to the prefrontal cortex was further highlighted by the finding in the opposite direction for the volume of the parietal neocortex, whose relationship with aging was best explained by a quadratic fit, with a curve ascendance from the end of the fourth decade of age onwards. Using samples of wider age ranges (from infancy to elderly life), two previous ROI-based MRI studies of aging have reported patterns of inverse relationship between age and prefrontal volumes that were best explained by linear models (Allen et al., 2005; Kennedy et al., in press). The discrepancy of our findings from the results of those investigations suggest that non-linearity of age-related prefrontal volume loss may be a specific feature of non-elderly adult life. This underscores the importance of incorporating non-linear approaches to morphometric MRI studies of healthy aging, in order to more directly address the nature of age-related variations in regional brain volumes across separate stages of life. However, since this is the first study to assess non-linear patterns of age-related GM changes during such specific period of human adulthood, the present results should be interpreted with caution, and further replication of our findings involving the prefrontal cortex is warranted. It is possible that our use of a population-based approach for the recruitment of study participants would have yielded results different from those obtained when convenience samples are employed (i.e. students and employees of the research institution and/or subjects recruited through advertisement, etc.) (Lee et al., 2007). Population-based designs are likely to reduce selection biases by ensuring that all individuals enrolled in the study have similar levels of exposure to environmental factors within the general population (Wacholder, 1995; Lee et al., 2007).

Both our whole-brain VBM search and the ROI mask-based analysis of regional volume estimates were indicative of a lack of GM volume decline in temporolimbic and anterior cingulate regions. These results are consistent with the view that limbic and paralimbic brain structures do not display the pattern of progressive GM shrinkage over the process of aging as seen in neocortical brain areas (Good et al., 2001; Gur et al., 2002; Grieve et al., 2005). In contrast, two large ROI-based MRI studies of healthy aging did describe significant age-related volumetric decrements in the hippocampus and/or parahippocampal gyrus (Allen et al., 2005; Kennedy et al., in press). However, the latter studies reported non-linearity in the relationship between temporolimbic volumes and age, with accelerated rates of GM loss appearing only from approximately 40–60 years of age onwards. It is possible to reconcile those findings with the results presented herein, as the upper age limit in our investigation was below the age range where accelerated rates of GM loss become apparent in those two MRI reports (Allen et al., 2005; Kennedy et al., in press). In our study, the curve-fitting analyses using the ROI-mask volume estimates showed that the relationship between temporolimbic volumes (corrected for global GM volumes) and age was best explained by a linear, ascending model. This mirrored the descending linear pattern of global GM volumes with aging, thus providing confirmation that the volumes of temporolimbic regions remained static over the adult age range covered in the present study.

Findings of age-related frontal neocortical volume reductions in adult life may be influenced by late processes of brain maturation. Such maturation changes involve increased myelination and synaptic pruning, both of which are associated with macroscopic reductions in GM volumes as assessed with MRI (Sowell et al., 2004). The diverging patterns of age-related GM changes in temporolimbic and paralimbic areas versus prefrontal regions in non-elderly, adult life are possibly related to differences in the time course of the myelination and synaptic pruning maturational processes in the brain. This is in accordance with the view that phylogenetically older brain areas mature earlier than higher order association cortices (Toga et al., 2006). The results of our study, taken together with previous findings of volume preservation of limbic structures with aging (Good et al., 2001; Gur et al., 2002; Grieve et al., 2005; Kalpouzos et al., 2009), provide support for the view that neurodegenerative changes related to aging are not present in limbic structures until advances stages of life, unless there is an intervening occurrence of specific neuropathological processes, such as those related to cerebrovascular disease risk (Gianaros et al., 2006) or Alzheimer's disease (Garrido et al., 2002). The relative resilience of temporolimbic and anterior cingulate regions against age-related brain changes in comparison to prefrontal cortical regions is compatible with the notion that the latter, being the last to mature, are more vulnerable to neurodegeneration due to their higher degree of plasticity (York and Steinberg, 1995; Toga et al., 2006).

One distinctive aspect of the present study is the absence of volume decrement over the aging process that we found bilaterally in the insula. It is important to highlight findings pertaining to this brain region, given its key relevance to emotional processing (Paulus and Stein, 2006) and the central representation of afferent visceral information (Critchley, 2005). Also, structural and/or functional abnormalities of the insula have been recently implicated in the pathophysiology of different adult-onset neuropsychiatric conditions, including anxiety disorders, mood disorders and schizophrenia (Job et al., 2002; Phillips et al., 2003b; Paulus and Stein, 2006). The findings reported herein are not consistent with those of previous VBM studies that investigated the relationship between GM volumes and aging into later decades of life, as these have found significant GM losses in the insula in association with age progression, in the same proportion as seen in neocortical regions (Abe et al., 2008; Good et al., 2001; Grieve et al., 2005; Kalpouzos et al., 2009). Thus it is possible to suggest that, during non-elderly adult life, the insula displays a pattern of no GM loss that is similar to that of limbic regions, but starts to show accelerated GM losses in later decades of life. The hypothesis of an intermediate profile of age-related volume changes in the insula is interesting in light of the fact that the GM surface of this brain region is composed of mesocortex, which shows a cytoarchitectural profile that is in the transition between the phylogenetically older allocortical structure of limbic regions and the isocortical profile that characterizes neocortical regions (Ribas, 2006). However, interpretations regarding age differences in the insula must be made with caution, as findings in this brain region may be particularly subject to artifacts due to limitations of the VBM approach (Kennedy et al., in press). Such VBM drawbacks include systematic registration errors during spatial normalization (Bookstein, 2001), particularly in the proximity of brain regions where there may be large differences in size and shape between groups, such as the lateral ventricles and major brain sulci (Duran et al., 2006; Kennedy et al., in press). Also, there may be biases in the segmentation process in brain areas where tissue contrast is poorly defined in MRI scans, and this may be particularly prevalent in the aging brain (Kennedy et al., in press). Finally, image smoothing with Gaussian filters may hamper the detection of between-group volumetric differences in small-sized brain regions, especially when large smoothing kernels are used (Uchida et al., 2008).

The above limitations of voxel-based approaches may explain discrepancies in the results of VBM and manual ROI-based MRI studies of healthy aging obtained not only in the insula, but also in other cortical brain regions. Such differences have been recently demonstrated in quantitative investigations that directly compared the two approaches (Kennedy et al., in press). Therefore, further MRI studies using the gold standard manual ROI approach are needed to confirm the overall pattern of VBM results reported herein in representative samples of healthy subjects, within the specific non-elderly age range as in the present study. Nevertheless, it should be noted that we also obtained estimates of regional brain volumes from the VBM data using ROI masks, an approach that has been shown to increase the agreement between VBM-derived and manual ROI volumetric data (Kennedy et al., in press). Our results with ROI masks extended the VBM finding of neocortical GM reductions with aging selectively in prefrontal cortical regions.

The sub-division of our sample by gender revealed different patterns of regional brain volumetric changes with aging in males and females across the age range studied. In male subjects, the whole-brain VBM analysis (with covariance for global GM changes) revealed clusters of accelerated GM decline in the neocortex and no GM loss in limbic/paralimbic regions, similarly to the patterns detected in the overall sample. Such region-specific significant linear correlations were absent in the VBM analyses for the female subgroup. Conversely, the analyses using ROI masks suggested that the lack of findings in the linear VBM analysis in the female subgroup was related to the fact that the pattern of brain aging in several brain regions in females was best represented by quadratic and cubic regression models, rather than a monotonic linear pattern. Specifically in the dorsolateral prefrontal cortex, volumetric changes in female subjects were restricted to the right hemisphere, where an ascending curve, probably related to a smaller decrement rate or even preservation of GM volume in this structure relative to the total amount of GM in brain, was observed until the fourth decade, followed by a more accelerated rate of GM volume decline from the fifth decade onwards. Also, non-linear patterns of GM change were detected in lateral temporal and parietal neocortices only in female subjects, indicating an increase in GM vulnerability to aging between 30 and 50 years of age. Among the factors that could possibly explain the differential regional brain vulnerability to normal aging between males and females, the influence of sex hormones has raised particular interest (Berman et al., 1997; Keenan et al., 2001; Fernandez et al., 2003; Eberling et al., 2004; Cowell et al., 2007). Several studies have indicated that sex hormones may play a role in the maintenance of temporolimbic and neocortical structural and functional integrity (Berman et al., 1997; Keenan et al., 2001; Fernandez et al., 2003; Eberling et al., 2004; Cowell et al., 2007). The lack of accelerated age-related neocortical atrophy in our subsample of non-elderly women (presumably premenopausal) may be at least in part linked to hormonal protection. It should be noted that the profile of gender distinctions in age effects reported herein has not been previously described in MRI studies of healthy aging. This may be related to differences in the age ranges evaluated in the present MRI study relative to earlier investigations (Murphy et al., 1996; Xu et al., 2000; Grieve et al., 2005; Benedetti et al., 2006; Carne et al., 2006; Cowell et al., 2007). In those previous cross-sectional MRI studies, the inclusion of children and adolescents on one side and elderly subjects on the other probably led to an overlapping of maturation and neurodegenerative processes, which would have been differentially influenced by hormonal effects across separate age ranges.

One methodological shortcoming of the present study is that we had to combine imaging data acquired using two different MRI scanners. However, the two scanners and acquisition protocols were identical, and we obtained high inter-equipment reliability indices for the neocortical and limbic regions that were the main focus of the investigation. Moreover, there were no differences in the patterns of significant linear correlations in those regions when the VBM analyses were repeated including scanner site as a confounding covariate. This indicated the validity of our strategy to combine data acquired with the two MRI scanners when investigating correlations between GM volumes and age in neocortical and limbic/paralimbic brain portions. However, the inter-scanner comparability of measurements for subcortical nuclei (i.e. the basal ganglia and thalamus) was less than optimal. Such methodological aspect could have influenced on the absence of significant negative correlations between age and regional GM in those structures, which have been reported in a proportion of previous MRI studies (Taki et al., 2004; Abe et al., 2008). However, there were no significant negative correlations between GM volume and age in these regions when we conducted analyses for the subsamples examined with each MRI equipment separately. This indicated that the absence of aging-related subcortical GM loss in our study was not due to scanner variability.

The present study has other methodological limitations that warrant caution in the interpretation of its results. We cannot discard the presence of underlying degenerative diseases, such as incipient Alzheimer's disease, in some of the older patients included in the sample; this might have influenced on the findings obtained, as subtle morphological changes can occur early over the course of Alzheimer's disease. Also, the reported between-gender differences in the non-linear patterns of age-related GM volumetric decline may have been influenced by the non-matching of females and males in regard to age, although there were not large discrepancies in the mean values and age range covered in each of those subgroups. Finally, it should be highlighted that this study employed a cross-sectional design, in which correlations between GM and age at specific time points are used to make inferences about how the aging process affect the brain structure over time. Therefore, replication of our findings is warranted in large longitudinal VBM studies involving the acquisition of serial MRI measurements over time in the same subject samples (Raz et al., 2005).

In conclusion, this cross-sectional, population-based VBM study provided evidence that, in healthy non-elderly individuals, there is a significant, non-linear pattern of age-related decline in GM volumes selectively in the prefrontal cortex, while the volumes of limbic, paralimbic and temporal neocortical areas remain largely unchanged. These findings provide further support for the hypothesis that the degree of vulnerability to neurodegenerative age-related changes across separate brain regions is inversely related to the phylogenetic origin of each region (i.e. the older the structure, the lower its vulnerability), thus resulting in a highly heterogeneous and asynchronous pattern of age-related morphometric brain changes. The mapping of such variability helps to increase knowledge about the maturational and degenerative processes that affect the healthy human brain across the span of life, and provides a framework that may improve our understanding about the patterns of progression of structural brain abnormalities that possibly occur in association with neuropsychiatric disorders.

## Conflict of interest

All authors declare to have no actual or potential conflicts of interest.

## Figures and Tables

**Table 1:** Voxel-based morphometry studies of age-related changes in regional gray matter volume in the healthy human brain.

Study	*n*	Age range	Age-related volume changes	
			Reduction	Preservation^a^
Good et al. (2001)	465	17–79	Pre and postcentral gyrus, anterior cingulate gyrus, angular gyrus, superior parietal gyrus and anterior insula bilaterally; right cerebellum (posterior lobe)	Hippocampus, amygdala, entorhinal cortex and thalamus bilaterally
Taki et al. (2004)	769	16–79	All cerebral cortex and basal ganglia	–
Grieve et al. (2005)	223	8–79	Middle frontal gyrus, pre and postcentral gyrus, and insula bilaterally; right inferior frontal gyrus	Hippocampus, amygdala, thalamus, inferior and middle temporal gyrus, and cingulate gyrus bilaterally
Alexander et al. (2006)	26	22–77	Frontal and temporal cortex bilaterally; right cerebellum	–
Kalpouzos et al. (2009)	45	20–83	Frontal, temporal and parietal cortex, insula and cerebellum bilaterally; left cingulate gyrus; right posterior hippocampus	Right anterior hippocampus and amygdala, thalamus bilaterally
Abe et al. (2008)	73	22–70	Frontal, temporal and parietal cortex bilaterally; left insula; right thalamus; left globus pallidus; left occipital cortex; cerebellum bilaterally	Bilateral cingulate gyrus
Kennedy et al. (in press)	200	18–81	Bilateral superior temporal gyrus and insula; left medial frontal gyrus; left pre-central and inferior frontal gyri,	

a Relative to the degree of whole-brain gray matter decrement.

**Table 2:** Demographic characteristics of the non-elderly adult individuals.

	Total sample (*n* = 89)	Males (*n* = 48)	Females (*n* = 41)	*p*
Mean age (±SD)	30.17 (±8.35)	27.83 (±7.26)	32.90 (±8.79)	0.004^a^

Age distribution of subjects (%)				
18–30	53 (59.6)	33 (68.8)	20 (48.8)	
31–40	23 (25.8)	12 (25.0)	11 (26.8)	
41–50	13 (14.6)	3 (6.2)	10 (24.4)	

Mean years of education (±SD)	10.02 (±4.08)	10.54 (±4.35)	9.41 (±3.71)	0.196^a^
Left handed subjects (%)	2 (2.2)	0 (0.0)	27 (65.9)	0.162^b^
Married subjects (%)	45 (50.6)	18 (37.5)	2 (4.9)	0.008^b^

SD = standard deviation.

a Student's *t*-test.

b Pearson's chi-squared test.

**Table 3:** Hypothesis-driven search for significant linear correlations between age and gray matter volumes in the overall sample of healthy individuals (*n* = 89, gender as nuisance variable), corrected for total gray matter brain volume.

Brain regions (SVC)^a^	Direction of significant correlation^b^	Hemisphere	Peak *Z*-scores^c^	BA^d^	Talairach and Tournoux coordinates (peak voxels)			*p* [FWE]^e^
					*x*	*y*	*z*	
Prefrontal cortex	Negative	Right	4.70 (Anterior middle frontal gyrus)	6	34	14	43	0.005
			4.28 (Posterior middle frontal gyrus)	46	40	49	6	0.018
		Left	-	–	–	–	–	–

Cerebellum	Negative	Right	-	–	–	–	–	–
		Left	4.25 (Posterior lobe, semi-lunar lobule)	–	−17	−63	−37	0.010

Cingulate gyrus	Positive	Right	4.39 (Anterior cingulate gyrus)	24	3	8	32	0.002
			5.03 (Posterior cingulate gyrus)	31	−1	−40	29	<0.001
		Left	4.03 (Anterior cingulate gyrus)	24	−1	10	30	0.010
			5.09 (Posterior cingulate gyrus)	31	−1	−40	29	<0.001

Temporolimbic region	Positive	Right	4.74 (Parahippocampal gyrus)	28	20	−17	−21	<0.001
		Left	4.80 (Amygdala)	–	−27	−3	−16	<0.001
			4.66 (Parahippocampal gyrus)	28	−20	−15	−22	<0.001
			3.97 (Hippocampus)	–	−29	−7	−24	0.007

Insula	Positive	Right	4.37 (Insula)	13	33	−17	11	0.001
		Left	4.95 (Insula)	13	−32	−1	−13	<0.001

Lateral temporal cortex	Positive	Right	3.84 (Superior temporal gyrus)	41	41	−34	9	0.036
		Left	4.69 (Superior temporal gyrus)	41	−41	−38	12	0.001

a Each region was circumscribed using the small volume correction (SVC) approach, with anatomically defined volume-of-interest masks.

b Negative correlations indicate age-related gray matter losses greater than the overall degree of gray matter decrement in the brain, while positive correlations indicate preservation relative to the overall degree of gray matter loss in the brain.

c *Z*-score for the voxels of peak statistical significance within each volume of interest (with the name of the corresponding anatomical brain structure in brackets).

d Approximate Brodmann areas.

e Family-wise error (FWE) correction for multiple comparisons at the level of individual voxels within the respective volume of interest.

**Table 4:** Hypothesis-driven search for significant linear correlations between age and gray matter volumes in the male subgroup (*n* = 48), corrected for total gray matter brain volume.

Brain regions (SVC)^a^	Direction of significant correlation^b^	Hemisphere	Peak *Z*-scores^c^	BA^d^	Talairach and Tournoux coordinates (peak voxels)			*p* [FWE]^e^
					*x*	*y*	*z*	
Prefrontal cortex	Negative	Right	4.71 (Middle frontal gyrus)	6	36	12	43	0.004
	Positive	Right	5.11 (Medial frontal gyrus)	25	10	12	−17	0.001
			4.12 (Inferior frontal gyrus)	47	24	20	−15	0.037
		Left	–	–	–	–	–	–

Cerebellum	Negative	Right	–	–	–	–	–	–
		Left	3.99 (Posterior lobe, semi-lunar lobule)	–	−22	−65	−35	0.031

Cingulate gyrus	Positive	Right	3.64 (Anterior cingulate gyrus)	24	1	28	13	0.040
			4.37 (Posterior cingulate gyrus)	24	3	−7	35	<0.001
		Left	4.10 (Anterior cingulate gyrus)	24	−3	30	15	0.009
			4.21 (Posterior cingulate gyrus)	24	−1	−19	34	0.006

Temporolimbic region	Positive	Right	5.38 (Parahippocampal gyrus)	28	17	−13	−19	<0.001
		Left	4.04 (Amygdala)	–	−27	1	−16	0.006

Insula	Positive	Right	4.90 (Insula)	13	33	−15	10	<0.001
		Left	3.94 (Insula)	13	−34	−13	1	0.008

Lateral temporal cortex	Positive	Right	3.83 (Transverse temporal gyrus)	41	40	−29	12	0.042
		Left	4.25 (Superior temporal gyrus)	22	−54	−25	0	0.008

a Each region was circumscribed using the small volume correction (SVC) approach, with anatomically defined volume-of-interest masks.

b Negative correlations indicate age-related gray matter losses greater than the overall degree of gray matter decrement in the brain, while positive correlations indicate preservation relative to the overall degree of gray matter loss in the brain.

c *Z*-score for the voxels of peak statistical significance within each volume of interest (with the name of the corresponding anatomical brain structure in brackets).

d Approximate Brodmann areas.

e Family-wise error (FWE) correction for multiple comparisons at the level of individual voxels within the respective volume of interest.

**Table 5:** Best fitting gray matter polynomial regression models by cerebral region, with values corrected for total gray matter brain volume (only regions with significant findings are shown).

Region of interest	Overall sample (*n* = 89)			Males (*n* = 48)			Females (n = 41)		
	Best fitting model	p*	*R*^2^	Best fitting model	p*	*R*^2^	Best fitting model	p*	*R*^2^
Right orbital frontal cortex	–	–	–	Quadratic/cubic	0.068	0.112	–	–	–
Left dorsolateral frontal cortex	Quadratic	0.002	0.135	Cubic	<0.001	0.342	–	–	–
Right dorsolateral frontal cortex	Cubic	<0.001	0.192	Cubic	<0.001	0.351	Cubic	0.038	0.202
Right dorsomedial frontal cortex	–	–	–	Quadratic	0.084	0.104	–	–	–
Left lateral temporal cortex	Linear	0.084	0.034	Linear	0.006	0.151	–	–	–
Right lateral temporal cortex	–	–	–	–	–	–	Quadratic	0.038	0.158
Right lateral parietal cortex	Quadratic	0.003	0.125	–	–	–	Quadratic	0.032	0.166
Right occipital cortex	Quadratic	0.089	0.055	–	–	–	–	–	–
Left insula	–	–	–	Linear	0.014	0.124	–	–	–
Right insula	–	–	–	Linear	0.013	0.127	–	–	–
Left anterior cingulate gyrus	–	–	–	Linear	0.030	0.098	–	–	–
Right anterior cingulate gyrus	–	–	–	Quadratic/cubic	0.029	0.145	–	–	–
Left temporolimbic region	Linear	0.002	0.102	Linear	0.001	0.217	Linear	0.028	0.118
Right temporolimbic region	Linear	<0.001	0.150	Linear	<0.001	0.342	–	–	–
Left cerebellum	Linear	0.028	0.054	–	–	–	Quadratic	0.076	0.127
Right cerebellum	Linear	0.085	0.034	–	–	–	–	–	–

* Significance level set at *p* < 0.05, with *p* < 0.10 values reported as trends.

## References

[bib1] Abe O., Yamasue H., Aoki S., Suga M., Yamada H., Kasai K., Masutani Y., Kato N., Kato N., Ohtomo K. (2008). Aging in the CNS: comparison of gray/white matter volume and diffusion tensor data. Neurobiol. Aging.

[bib2] Adler C.M., DelBello M.P., Jarvis K., Levine A., Adams J., Strakowski S.M. (2007). Voxel-based study of structural changes in first-episode patients with bipolar disorder. Biol. Psychiatry.

[bib3] Alexander G.E., Chen K., Merkley T.L., Reiman E.M., Caselli R.J., Aschenbrenner M., Santerre-Lemmon L., Lewis D.J., Pietrini P., Teipel S.J., Hampel H., Rapoport S.I., Moeller J.R. (2006). Regional network of magnetic resonance imaging gray matter volume in healthy aging. Neuroreport.

[bib4] Allen J.S., Bruss J., Brown C.K., Damasio H. (2005). Normal neuroanatomical variation due to age: the major lobes and a parcellation of the temporal region. Neurobiol. Aging.

[bib5] Benedetti B., Charil A., Rovaris M., Judica E., Valsasina P., Sormani M.P., Filippi M. (2006). Influence of aging on brain gray and white matter changes assessed by conventional, MT, and DT MRI. Neurology.

[bib6] Berman K.F., Schmidt P.J., Rubinow D.R., Danaceau M.A., Van Horn J.D., Esposito G., Ostrem J.L., Weinberger D.R. (1997). Modulation of cognition-specific cortical activity by gonadal steroids: a positron-emission tomography study in women. Proc. Natl. Acad. Sci. U.S.A..

[bib7] Bookstein F.L. (2001). “Voxel-based morphometry” should not be used with imperfectly registered images. Neuroimage.

[bib8] Brett M., Johnsrude I.S., Owen A.M. (2002). The problem of functional localization in the human brain. Nat. Rev. Neurosci..

[bib9] Carne R.P., Vogrin S., Litewka L., Cook M.J. (2006). Cerebral cortex: an MRI-based study of volume and variance with age and sex. J. Clin. Neurosci..

[bib10] Cowell P.E., Sluming V.A., Wilkinson I.D., Cezayirli E., Romanowski C.A., Webb J.A., Keller S.S., Mayes A., Roberts N. (2007). Effects of sex and age on regional prefrontal brain volume in two human cohorts. Eur. J. Neurosci..

[bib11] Critchley H.D. (2005). Neural mechanisms of autonomic, affective, and cognitive integration. J. Comp. Neurol..

[bib12] Duran, F.L.S., Valente, A.A., Miguel, E.C., Castro, C.C., Busatto, G.F., 2006. Risk of artifacts due to enlarged ventricles using voxel-based morphometry studies. Poster presented at Organization for Human Brain Mapping conference, Florence, IT. Neuroimage (Suppl.), 31.

[bib13] Eberling J.L., Wu C., Tong-Turnbeaugh R., Jagust W.J. (2004). Estrogen- and tamoxifen-associated effects on brain structure and function. Neuroimage.

[bib14] Fernandez G., Weis S., Stoffel-Wagner B., Tendolkar I., Reuber M., Beyenburg S., Klaver P., Fell J., de Greiff A., Ruhlmann J., Reul J., Elger C.E. (2003). Menstrual cycle-dependent neural plasticity in the adult human brain is hormone, task, and region specific. J. Neurosci..

[bib15] First M.B., Spitzer R.L., Gibbon M., Williams J.B.W. (1995). Structured Clinical Interview for DSM-IV Axis I Disorders—Patient Edition (SCID-I/P).

[bib16] Friston K.J., Holmes A.P., Worsley K.J., Poline J.P., Frith C.D., Frackowiak R.S.J. (1994). Statistic parametric maps in functional imaging: a general linear approach. Hum. Brain Mapp..

[bib17] Gallagher M., Schoenbaum G. (1999). Functions of the amygdala and related forebrain areas in attention and cognition. Ann. N.Y. Acad. Sci..

[bib18] Garrido G.E., Furuie S.S., Buchpiguel C.A., Bottino C.M., Almeida O.P., Cid C.G., Camargo C.H., Castro C.C., Glabus M.F., Busatto G.F. (2002). Relation between medial temporal atrophy and functional brain activity during memory processing in Alzheimer's disease: a combined MRI and SPECT study. J. Neurol. Neurosurg. Psychiatry.

[bib19] Gianaros P.J., Greer P.J., Ryan C.M., Jennings J.R. (2006). Higher blood pressure predicts lower regional grey matter volume: consequences on short-term information processing. Neuroimage.

[bib20] Good C.D., Johnsrude I.S., Ashburner J., Henson R.N., Friston K.J., Frackowiak R.S. (2001). A voxel-based morphometric study of ageing in 465 normal adult human brains. Neuroimage.

[bib21] Grieve S.M., Clark C.R., Williams L.M., Peduto A.J., Gordon E. (2005). Preservation of limbic and paralimbic structures in aging. Hum. Brain Mapp..

[bib22] Gur R.C., Gunning-Dixon F.M., Turetsky B.I., Bilker W.B., Gur R.E. (2002). Brain region and sex differences in age association with brain volume: a quantitative MRI study of healthy young adults. Am. J. Geriatr. Psychiatry.

[bib23] Honea R., Crow T.J., Passingham D., Mackay C.E. (2005). Regional deficits in brain volume in schizophrenia: a meta-analysis of voxel-based morphometry studies. Am. J. Psychiatry.

[bib24] Jernigan T.L., Archibald S.L., Fennema-Notestine C., Gamst A.C., Stout J.C., Bonner J., Hesselink J.R. (2001). Effects of age on tissues and regions of the cerebrum and cerebellum. Neurobiol. Aging.

[bib25] Job D.E., Whalley H.C., McConnell S., Glabus M., Johnstone E.C., Lawrie S.M. (2002). Structural gray matter differences between first-episode schizophrenics and normal controls using voxel-based morphometry. Neuroimage.

[bib26] Kalpouzos G., Chetelat G., Baron J.C., Landeau B., Mevel K., Godeau C., Barre L., Constans J.M., Viader F., Eustache F., Desgranges B. (2009). Voxel-based mapping of brain gray matter volume and glucose metabolism profiles in normal aging. Neurobiol. Aging.

[bib27] Keenan P.A., Ezzat W.H., Ginsburg K., Moore G.J. (2001). Prefrontal cortex as the site of estrogen's effect on cognition. Psychoneuroendocrinology.

[bib28] Kennedy, K.M., Erickson, K.I., Rodrigue, K.M., Voss, M.W., Colcombe, S.J., Kramer, A.F., Acker, J.D., Raz, N. Age-related differences in regional brain volumes: A comparison of optimized voxel-based morphometry to manual volumetry. Neurobiol. Aging, in press. 10.1016/j.neurobiolaging.2007.12.020 PMC2756236 18276037

[bib29] Kumaran D., Maguire E.A. (2005). The human hippocampus: cognitive maps or relational memory?. J. Neurosci..

[bib30] Lee W., Bindman J., Ford T., Glozier N., Moran P., Stewart R., Hotopf M. (2007). Bias in psychiatric case–control studies: literature survey. Br. J. Psychiatry.

[bib31] Lemaitre H., Crivello F., Grassiot B., Alperovitch A., Tzourio C., Mazoyer B. (2005). Age- and sex-related effects on the neuroanatomy of healthy elderly. Neuroimage.

[bib32] Mayberg H.S. (2003). Modulating dysfunctional limbic–cortical circuits in depression: towards development of brain-based algorithms for diagnosis and optimized treatment. Br. Med. Bull..

[bib33] Menezes P.R., Johnson S., Thornicroft G., Marshall J., Prosser D., Bebbington P., Kuipers E. (1996). Drug and alcohol problems among individuals with severe mental illness in south London. Br. J. Psychiatry.

[bib34] Mu Q., Xie J., Wen Z., Weng Y., Shuyun Z. (1999). A quantitative MR study of the hippocampal formation, the amygdala, and the temporal horn of the lateral ventricle in healthy subjects 40 to 90 years of age. AJNR Am. J. Neuroradiol..

[bib35] Murphy D.G., DeCarli C., McIntosh A.R., Daly E., Mentis M.J., Pietrini P., Szczepanik J., Schapiro M.B., Grady C.L., Horwitz B., Rapoport S.I. (1996). Sex differences in human brain morphometry and metabolism: an in vivo quantitative magnetic resonance imaging and positron emission tomography study on the effect of aging. Arch. Gen. Psychiatry.

[bib36] Paulus M.P., Stein M.B. (2006). An insular view of anxiety. Biol. Psychiatry.

[bib37] Phillips M.L., Drevets W.C., Rauch S.L., Lane R. (2003). Neurobiology of emotion perception. I: The neural basis of normal emotion perception. Biol. Psychiatry.

[bib38] Phillips M.L., Drevets W.C., Rauch S.L., Lane R. (2003). Neurobiology of emotion perception. II: Implications for major psychiatric disorders. Biol. Psychiatry.

[bib39] Pieperhoff P., Homke L., Schneider F., Habel U., Shah N.J., Zilles K., Amunts K. (2008). Deformation field morphometry reveals age-related structural differences between the brains of adults up to 51 years. J. Neurosci..

[bib40] Pribram K.H. (2006). What makes humanity humane. J. Biomed. Discov. Collab..

[bib41] Raz N., Rodrigues K.M., Kennedy K.M., Head D., Gunning-Dixon F., Acker J.D. (2003). Differential aging of the human striatum: longitudinal evidence. AJNR Am. J. Neuroradiol..

[bib42] Raz N., Lindenberger U., Rodrigue K.M., Kennedy K.M., Head D., Williamson A., Dahle C., Gerstorf D., Acker J.D. (2005). Regional brain changes in aging healthy adults: general trends, individual differences and modifiers. Cereb. Cortex.

[bib43] Resnick S.M., Pham D.L., Kraut M.A., Zonderman A.B., Davatzikos C. (2003). Longitudinal magnetic resonance imaging studies of older adults: a shrinking brain. J. Neurosci..

[bib44] Ribas G.C. (2006). Considerations about the nervous system phylogenetic evolution, behavior, and the emergence of consciousness. Rev. Bras. Psiquiatr..

[bib45] Saunders J.B., Aasland O.G., Babor T.F., de la Fuente J.R., Grant M. (1993). Development of the Alcohol Use Disorders Identification Test (AUDIT): WHO Collaborative Project on Early Detection of Persons with Harmful Alcohol Consumption—II. Addiction.

[bib46] Schaufelberger M.S., Duran F.L.S., Lappin J.M., Scazufca M., Amaro A., Leite C.C., de Castro C.C., Murray R.M., McGuire P.K., Menezes P.R., Busatto G.F. (2007). Grey matter abnormalities in Brazilians with first-episode psychosis. Br. J. Psychiatry.

[bib47] Sowell E.R., Thompson P.M., Holmes C.J., Jernigan T.L., Toga A.W. (1999). In vivo evidence for post-adolescent brain maturation in frontal and striatal regions. Nat. Neurosci..

[bib48] Sowell E.R., Thompson P.M., Tessner K.D., Toga A.W. (2001). Mapping continued brain growth and gray matter density reduction in dorsal frontal cortex: inverse relationships during postadolescent brain maturation. J. Neurosci..

[bib49] Sowell E.R., Peterson B.S., Thompson P.M., Welcome S.E., Henkenius A.L., Toga A.W. (2003). Mapping cortical change across the human life span. Nat. Neurosci..

[bib50] Sowell E.R., Thompson P.M., Toga A.W. (2004). Mapping changes in the human cortex throughout the span of life. Neuroscientist.

[bib51] Sullivan E.V., Marsh L., Mathalon D.H., Lim K.O., Pfefferbaum A. (1995). Age-related decline in MRI volumes of temporal lobe gray matter but not hippocampus. Neurobiol. Aging.

[bib52] Taki Y., Goto R., Evans A., Zijdenbos A., Neelin P., Lerch J., Sato K., Ono S., Kinomura S., Nakagawa M., Sugiura M., Watanabe J., Kawashima R., Fukuda H. (2004). Voxel-based morphometry of human brain with age and cerebrovascular risk factors. Neurobiol. Aging.

[bib53] Talairach J., Tournoux P. (1988). Coplanar Stereotactic Atlas of the Human Brain.

[bib54] Tisserand D.J., Visser P.J., van Boxtel M.P., Jolles J. (2000). The relation between global and limbic brain volumes on MRI and cognitive performance in healthy individuals across the age range. Neurobiol. Aging.

[bib55] Tisserand D.J., Pruessner J.C., Sanz Arigita E.J., van Boxtel M.P., Evans A.C., Jolles J., Uylings H.B. (2002). Regional frontal cortical volumes decrease differentially in aging: an MRI study to compare volumetric approaches and voxel-based morphometry. Neuroimage.

[bib56] Toga A.W., Thompson P.M., Mori S., Amunts K., Zilles K. (2006). Towards multimodal atlases of the human brain. Nat. Rev. Neurosci..

[bib57] Uchida R.R., Del-Ben C.M., Araújo D., Busatto-Filho G., Duran F.L., Crippa J.A. (2008). Correlation between voxel based morphometry and manual volumetry in magnetic resonance images of the human brain. An. Acad. Bras. Cienc..

[bib58] Wacholder S. (1995). Design issues in case–control studies. Stat. Methods Med. Res..

[bib59] Xu J., Kobayashi S., Yamaguchi S., Iijima K., Okada K., Yamashita K. (2000). Gender effects on age-related changes in brain structure. AJNR Am. J. Neuroradiol..

[bib60] York G.K., Steinberg D.A. (1995). Hughlings Jackson's theory of recovery. Neurology.

